# Supplementary material for: Lichen-Derived Diffractaic Acid Inhibited Dengue Virus Replication in a Cell-Based System
Source: Molecules. 2023 Jan 18;28(3):974. doi: 10.3390/molecules28030974 (PMC9918999; doi:10.3390/molecules28030974)
Supplement: Supplementary file 1 [file molecules-28-00974-s001.zip › molecules-2124065-supplementary.pdf]

Supporting information

Lichen-derived diffractaic acid inhibited dengue virus replication in a cell-based system

Naphat Loeanurit [1, 2], Truong Lam Tuong [3,4], Van-Kieu Nguyen [3,5,6], Vipanee Vibulakhaophan [1,7], Kowit Hengpha-satporn [8], Yasuteru Shigeta [8], Si Xian Ho [9], Justin Jang Hann Chu [9], Thanyada Rungrotmongkol [10,11], Warinthorn Chavasiri [3] & Siwaporn Boonyasuppayakorn [1,*]

1 Center of Excellence in Applied Medical Virology, Department of Microbiology, Faculty of Medicine, Chulalongkorn University, Bangkok, 10330, Thailand
2 Interdisciplinary Program in Microbiology, Graduate School, Chulalongkorn University, Bangkok, 10330, Thailand
3 Center of Excellence in Natural Products Chemistry, Department of Chemistry, Faculty of Science, Chulalongkorn University, Bangkok, 10330, Thailand
4 Department of Medicinal Chemistry, Faculty of Pharmacy, University of Medicine and Pharmacy at Ho Chi Minh City, Ho Chi Minh City 700000 Vietnam
5 Institute of Fundamental and Applied Sciences, Duy Tan University, Ho Chi Minh City 710000, Vietnam
6 Faculty of Natural Sciences, Duy Tan University, Da Nang 550000, Vietnam
7 Department of Biology, Faculty of Science, Chulalongkorn University, Bangkok, 10330, Thailand
8 Center for Computational Sciences, University of Tsukuba, 1-1-1 Tennodai, Tsukuba, Ibaraki 305-8577, Japan
9 Laboratory of Molecular RNA Virology and Antiviral Strategies, Department of Microbiology and Immunology, Yong Loo Lin School of Medicine, National University of Singapore, Singapore 117545, Singapore
10 Program in Bioinformatics and Computational Biology, Graduate School, Chulalongkorn University, Bangkok 10330, Thailand
11 Biocatalyst and Environmental Biotechnology Research Unit, Department of Biochemistry, Faculty of Science, Chulalongkorn University, Bangkok 10330, Thailand
* Correspondence: siwaporn.b@chula.ac.th

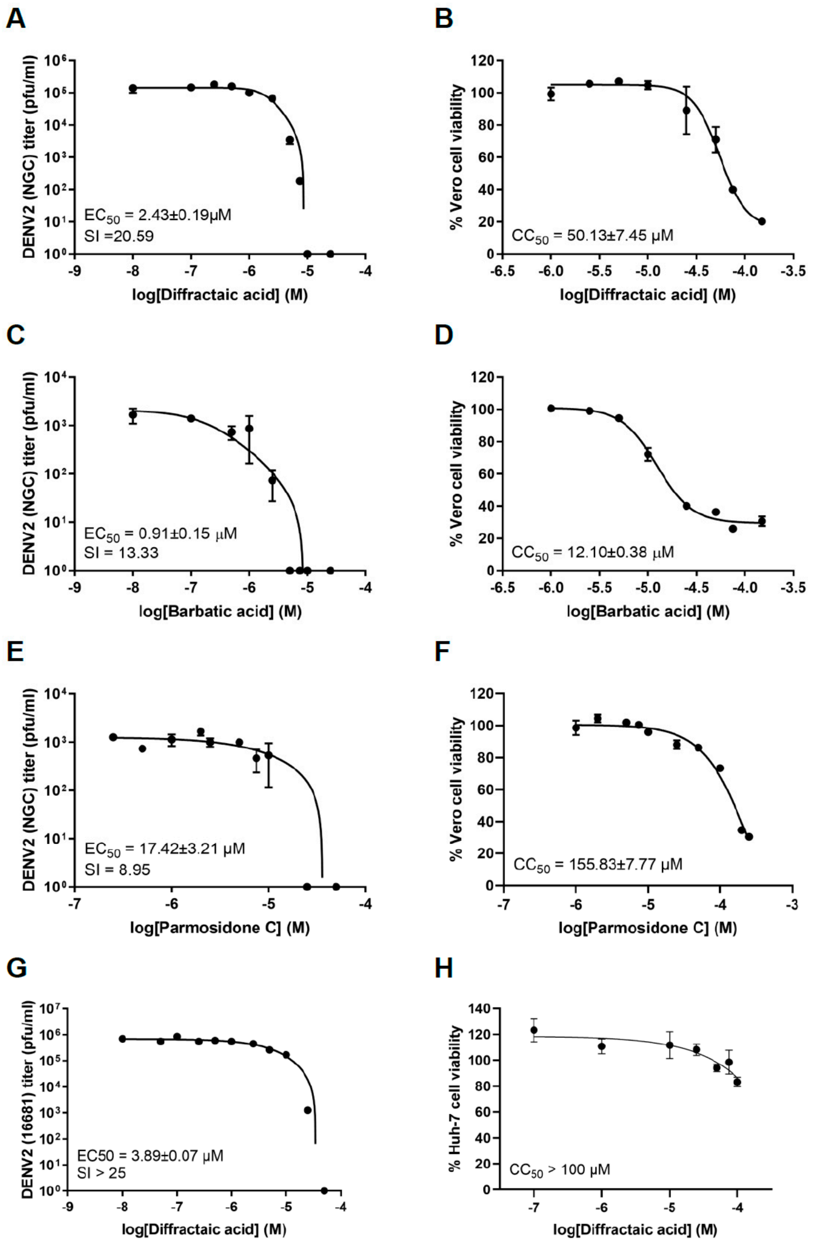

Supplementary Figure S1. Efficacies and cytotoxicities of (**A**,**B**) diffractaic acid, (**C**,**D**) barbatic acid, and (**E**,**F**) parmosidone C, against DENV2 NGC in Vero cells, as well as the efficacies and cytotoxicities of (**G**,**H**) diffractaic acid against DENV2 16881 in Huh-7 cells. Each graph represents one of three independent experiments. Data represents mean ± standard error of the mean from three independent experiments and the selectivity index was calculated from the (SI = CC_{50}/EC_{50} ratio).

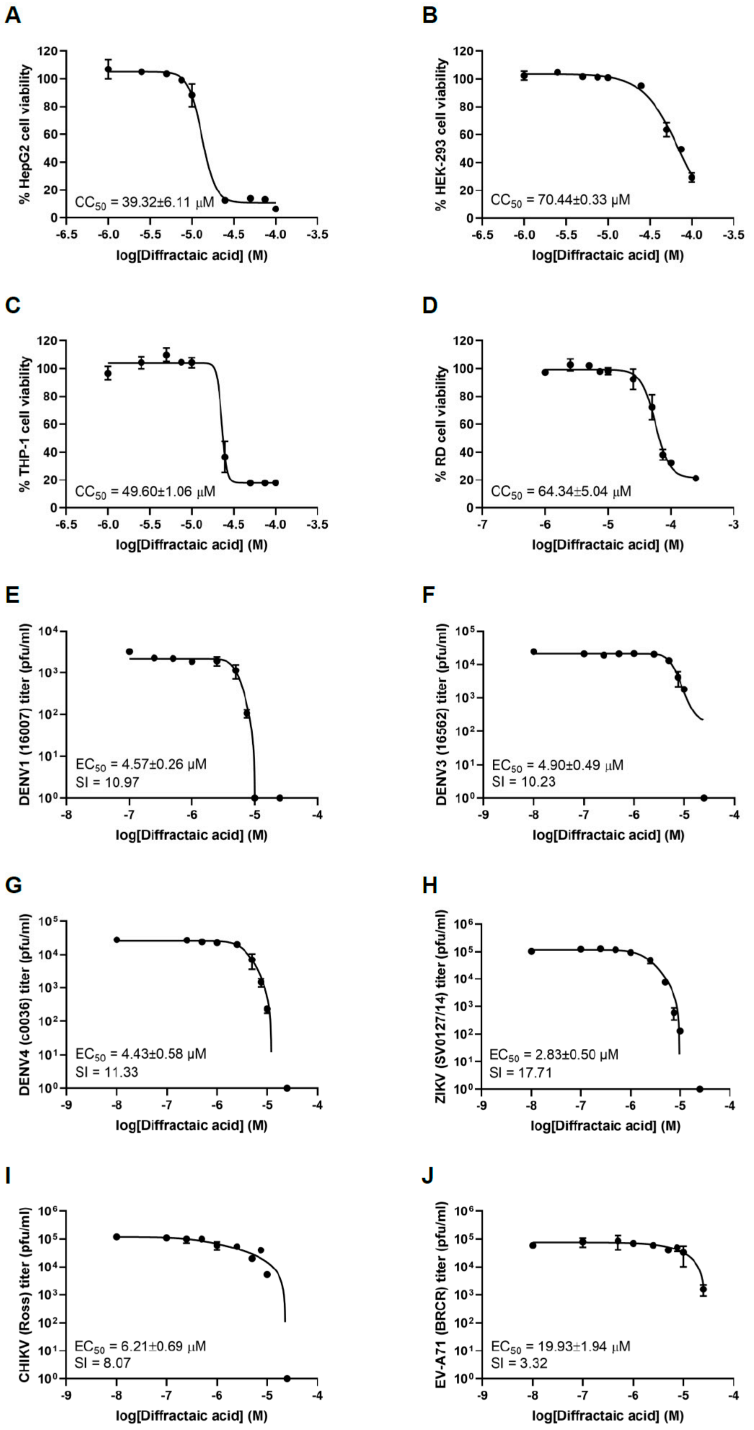

Supplementary Figure S2. (**A–D**) Cytotoxicities and (**E–J**) efficacies of diffractaic acid in various cells and viruses. Cytotoxicities at 48 h in (**A**) HepG2, (**B**) HEK-293, (**C**) THP-1 and (**D**) RD cells, respectively were examined by MTS assays and efficacy against (**E**) DENV1, (**F**) DENV 3, (**G**) DENV4, (**H**) ZIKV, (**I**) CHIKV, and (**J**) EV-A71 respectively were examined by plaque titration. Each dose-response curve represents one of three independent experiments. Results were means ± stand error of the means from three independent experiments.

Fusion inhibition assay

Fusion inhibition assay was performed for inhibition of the conformational change of DENV envelope and initiate phospholipid membrane fusion Syncytial formation under acidic condition (pH 5-6) (1). Diffractaic acid was added to DENV2 NGC -infected C6/36 cells (M.O.I. of 1) and incubated for 2 days. 4G2 and DMSO were used as positive and no inhibition control. Syncytial formation was induced by adding 0.5 M MES (pH 5-6) to induced acidic condition and incubated for 24-48 hours. The result showed that the compound did not prevent cell-to-cell fusion (Figure S3). Therefore, it was unlikely that a DENV envelope protein would be a molecular target of diffractaic acid.

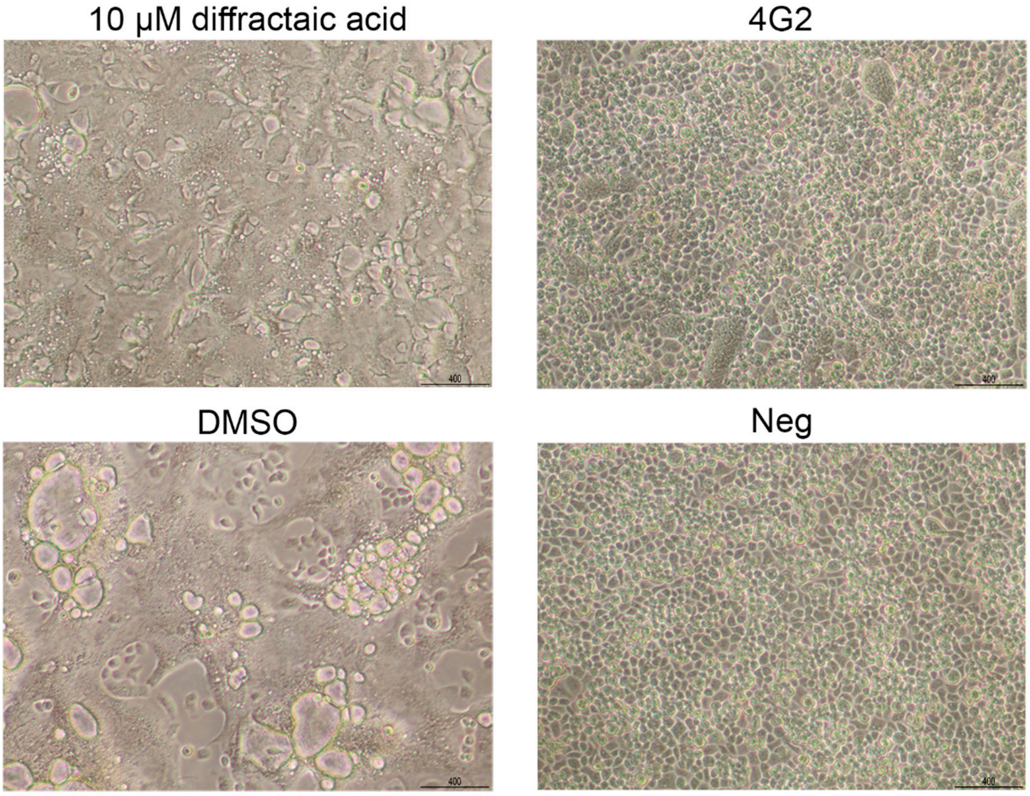

Supplementary Figure S3. Fusion inhibition assay. C6/36 cells were infected with DENV2 NGC (M.O.I. of 1) followed by the addition of compound. 4G2 and DMSO were used as positive and no inhibition control. After 2 days of incubation, 0.5 M MES was added to induce cell fusion. Pictures were taken after 0.5 M MES was added for 1-2 days until fused cells were observed under the light microscope.

Whole blot from SDS-PAGE and Western blot analysis of DENV E protein and β-actin

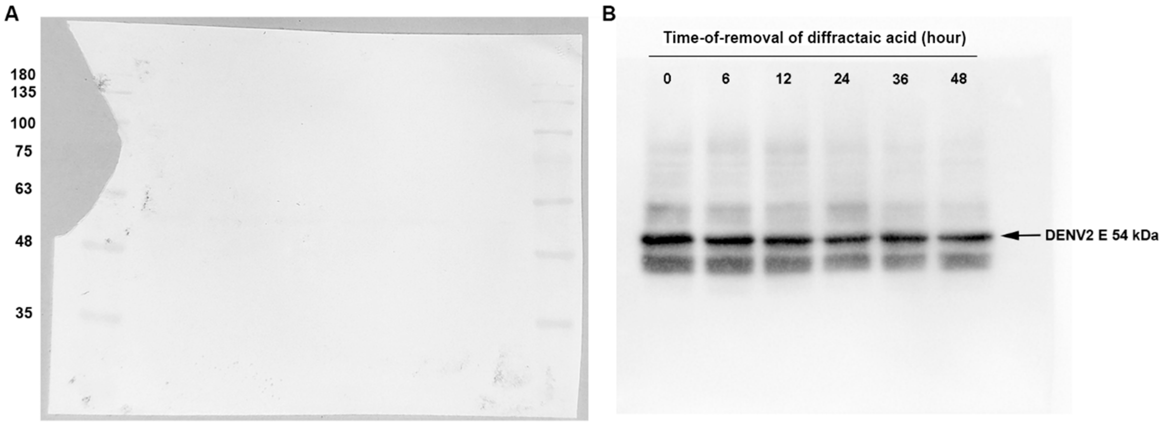

Supplementary Figure S4. Whole blot from time-of-removal assay of diffractaic acid for DENV2 E protein.

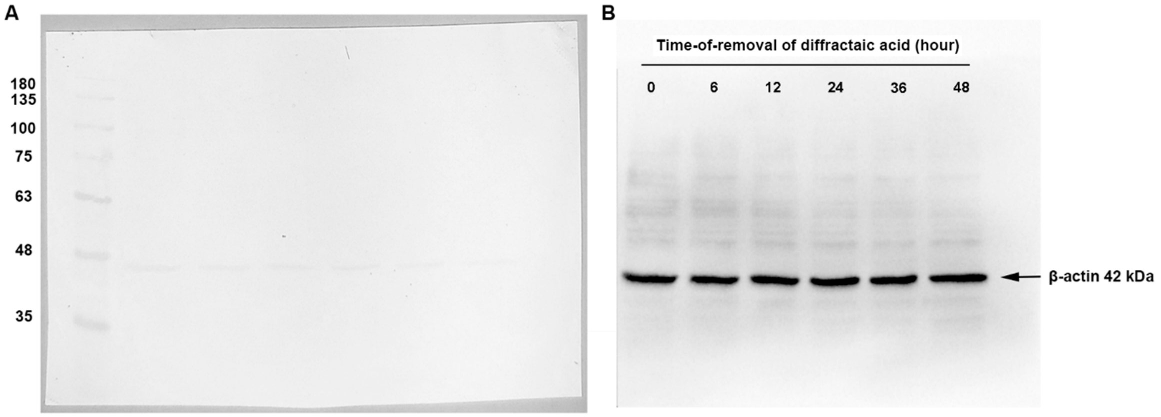

Supplementary Figure S5. Whole blot from time-of-removal assay of diffractaic acid for β-actin.

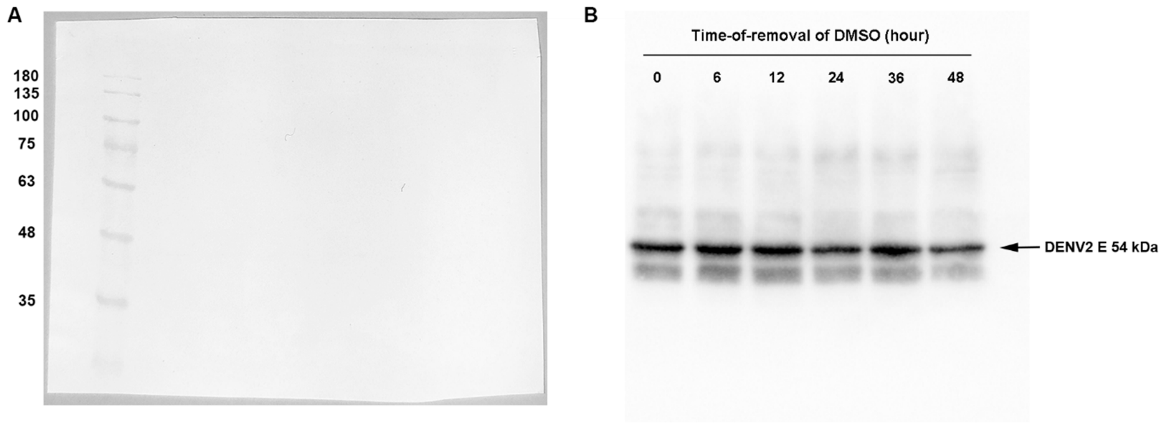

Supplementary Figure S6. Whole blot from time-of-removal assay of DMSO for DENV2 E protein.

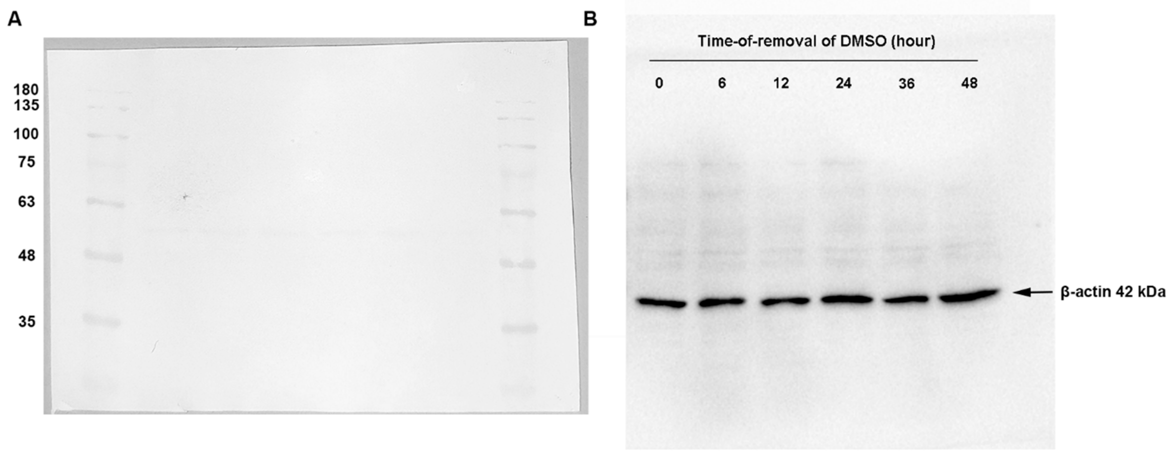

Supplementary Figure S7. Whole blot from time-of-removal assay of DMSO for β-actin.

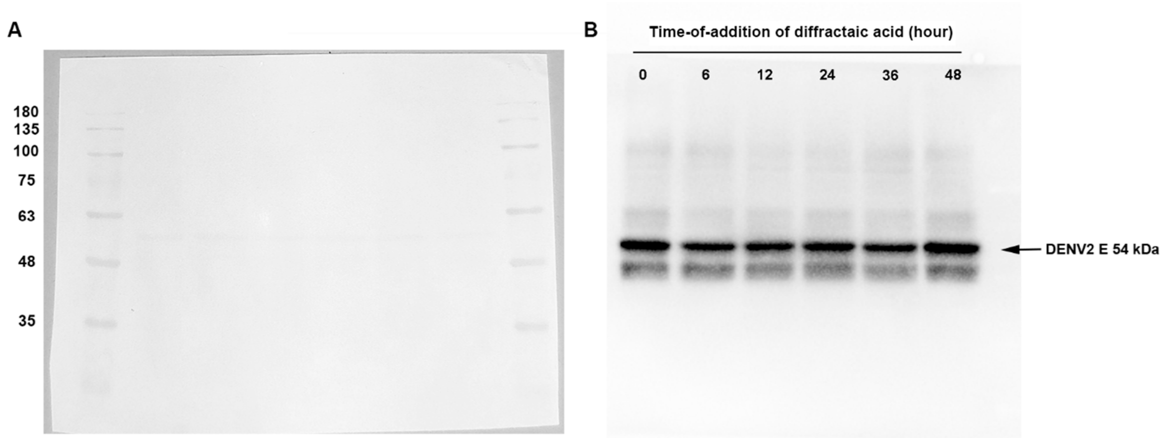

Supplementary Figure S8. Whole blot from time-of-addition assay of diffractaic acid for DENV2 E protein.

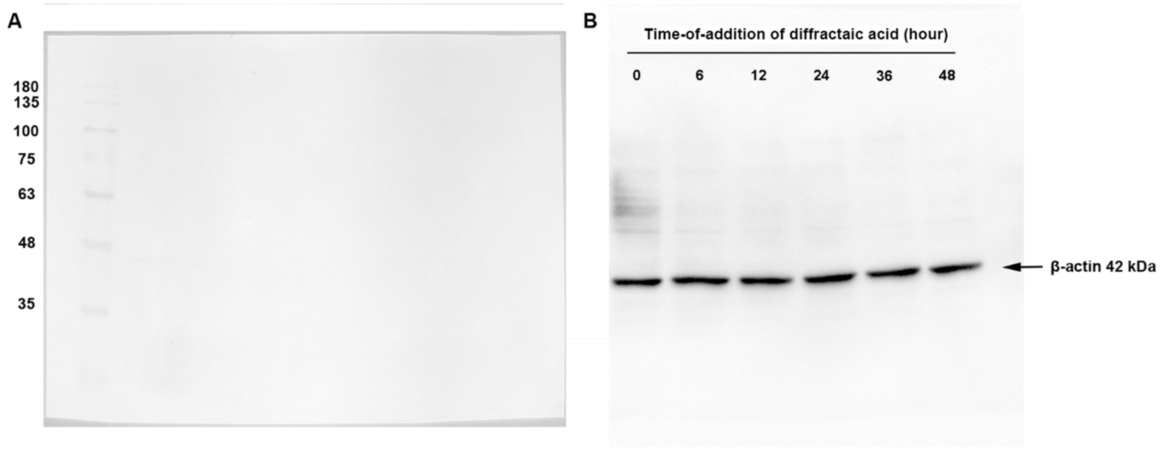

Supplementary Figure S9. Whole blot from time-of-addition assay of diffractaic acid for β-actin.

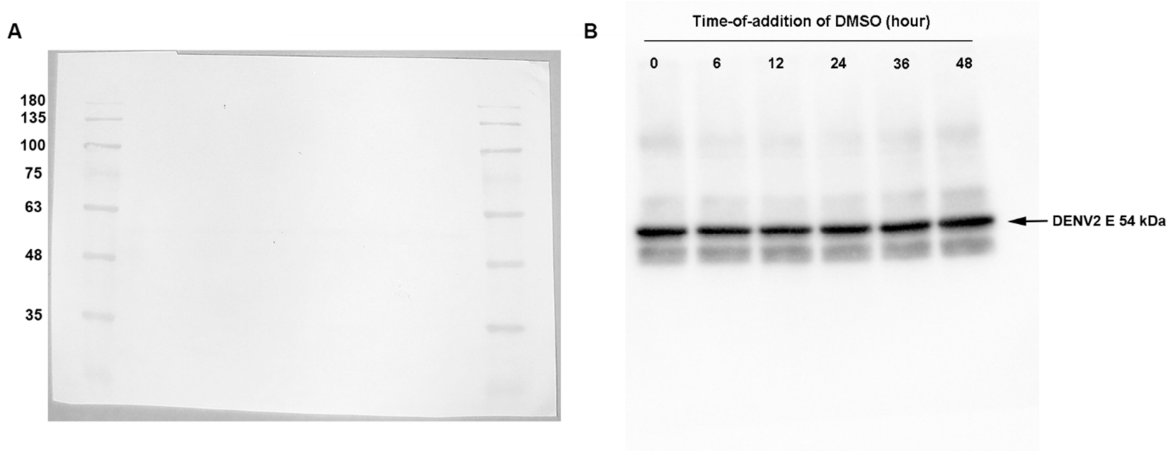

Supplementary Figure S10. Whole blot from time-of-addition assay of DMSO for DENV2 E protein**.**

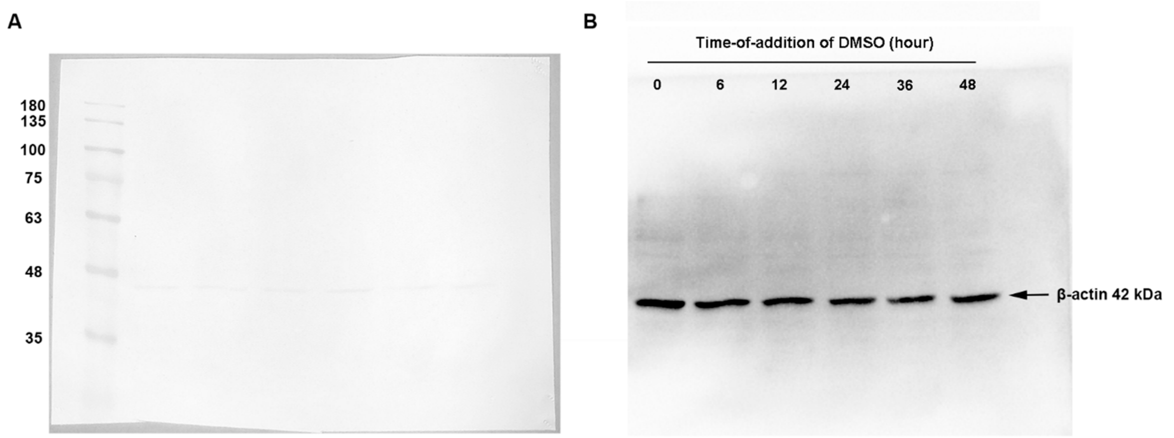

Supplementary Figure S11. Whole blot from time-of-addition assay of DMSO for β-actin.

Determination of the dynamic effect of diffractaic acid on a single round of DENV2 infection

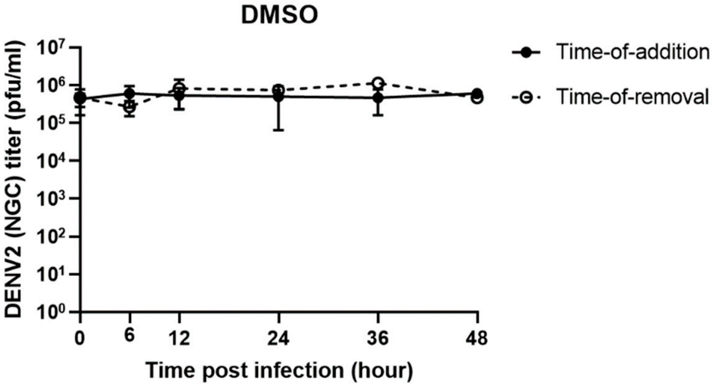

Supplementary Figure S12. Time-of-addition and time-of-removal assay of DENV2-infected Vero cells (M.O.I. of 1). Supernatants of DMSO-treated samples were collected for DENV2 plaque titration.

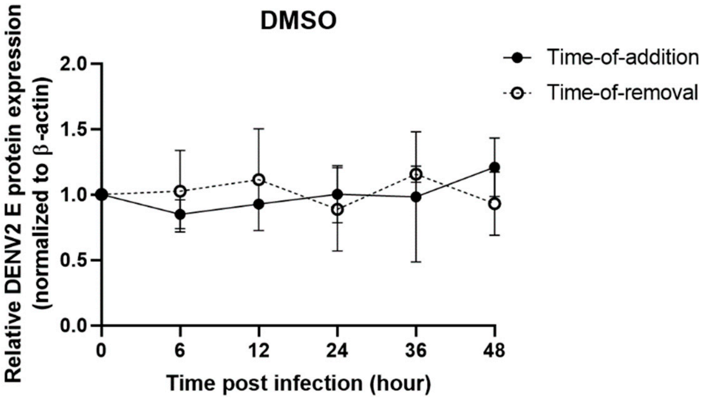

Supplementary Figure S13. Relative DENV2 E protein expression of DMSO-treated Vero cells.

Generation of compound resistant mutant

Huh-7 cells were infected with DENV2 16681 (M.O.I. of 1) for 1 hour at 37°C under 5% CO2. After infection, cells were washed with PBS. The compound at designated concentrations or 1% DMSO was added to the virus-infected cells and maintained for 2-3 days or until the 50% cytopathic effect was observed. Supernatants were collected for subsequent infection in Huh-7 cells and analyzed by plaque titration assay. The viral samples from compound-treated and DMSO-treated groups at passage 15th were collected for RNA extraction. The sequences were analyzed by next generation sequencing. Only one single nucleotide substitution (G5178A) was found from diffractaic acid treatment.

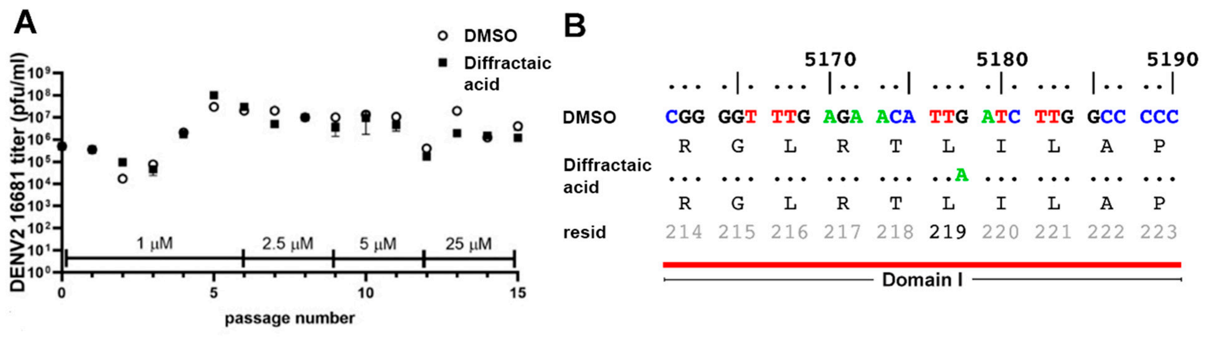

Supplementary Figure S14. Generation of diffractaic acid resisted DENV2. (**A**) DENV2 16681 titer of each infected cell passage treated with diffractaic acid compared to DMSO as control. (**B**) A single nucleotide substitution (G5178A) was discovered from diffractaic acid treatment compared with DMSO wildtype, caused a silent mutation of leucine at L219.
